# Trends in Sentinel Lymph Node Biopsies in Patients With Inflammatory Breast Cancer in the US

**DOI:** 10.1001/jamanetworkopen.2021.48021

**Published:** 2022-02-11

**Authors:** Alan Sosa, Xiudong Lei, Wendy A. Woodward, Mariana Chavez Mac Gregor, Anthony Lucci, Sharon H. Giordano, Kevin T. Nead

**Affiliations:** 1 Department of Radiation Oncology, University of Texas MD Anderson Cancer Center, Houston; 2 Department of Health Services Research, University of Texas MD Anderson Cancer Center, Houston; 3 Department of Breast Medical Oncology, University of Texas MD Anderson Cancer Center, Houston; 4 Department of Breast Surgical Oncology, University of Texas MD Anderson Cancer Center, Houston; 5 Department of Epidemiology, University of Texas MD Anderson Cancer Center, Houston

## Abstract

**Importance:**

The standard of care for inflammatory breast cancer (IBC) is neoadjuvant chemotherapy, total mastectomy with axillary lymph node dissection (ALND), and postmastectomy radiation therapy. Existing studies suggest that sentinel lymph node biopsy (SLNB) may not be reliable in IBC. The use and frequency of SLNB in women with IBC is not well characterized.

**Objective:**

To determine the frequency and temporal trend of SLNB in patients with IBC.

**Design, Setting, and Participants:**

This retrospective cohort study used the National Cancer Database, a nationwide hospital-based cancer registry, and included women who were diagnosed with nonmetastatic IBC and underwent axillary surgery from 2012 to 2017. Data were analyzed from January 2021 to May 2021.

**Exposures:**

Any SLNB, including SLNB alone and SLNB followed by ALND, and ALND alone.

**Main Outcomes and Measures:**

Scatterplot fit with a linear regression model were used to evaluate the yearly increase of any SLNB use. Multivariable logistic regression models to evaluate the association of study variables with the outcome of any SLNB.

**Results:**

This study included a total of 1096 women (mean [SD] age, 56.1 [12.9] years) who were 18 years or older with nonmetastatic IBC diagnosed between 2012 and 2017. Of the 186 of 1096 women (17%) who received any SLNB, 137 (73.7%) were White individuals; and of the 910 of 1096 women (83%) who received an ALND only, 676 (74.3%) were White individuals. Among women undergoing any SLNB, 119 of 186 (64%) did not undergo a completion ALND. There was a statistically significant increasing trend in the use of SLNB from 2012 to 2017 (22 of 205 patients [11%] vs 32 of 148 patients [22%]; *P* = .004). In multivariable analysis, the use of SLNB was associated with diagnosis year (2017 vs 2012; odds ratio [OR], 2.26; 95% CI, 1.26-4.20), clinical nodal status (cN3 vs 0; OR, 0.39; 95% CI, 0.22-0.67), and receipt of reconstructive surgery (OR, 1.80; 95% CI, 1.09-2.96).

**Conclusions and Relevance:**

The findings of this cohort study suggest that there is frequent and increasing use of SLNB in patients with IBC that is not evidence-based or supported by current treatment guidelines.

## Key Points

**Question:**

What is the sentinel lymph node biopsy (SLNB) rate in women with inflammatory breast cancer (IBC)?

**Findings:**

In this cohort study of 1096 women with IBC, the use of SLNB increased during the study period from 11% in 2012 to 22% in 2017.

**Meaning:**

This study found increasing use of SLNB for patients with IBC that is not evidence-based.

## Teaser

This cohort study evaluates the frequency and temporal trend of sentinel lymph node biopsy among US women with inflammatory breast cancer.

## Introduction

Inflammatory breast cancer (IBC) is an aggressive type of cancer accounting for 1% to 6% of all breast cancers diagnosed in the US per year.^1^ IBC is diagnosed based on clinical criteria secondary to acute inflammatory changes in the breast resulting in diffuse erythema and edema from the obstruction of dermal lymphatic channels with tumor emboli.^2^ Up to 90% of patients with IBC may have regional nodal disease at presentation with a 2-fold higher mortality rate than noninflammatory locally advanced breast cancer.^3,4^

The standard of care for IBC is trimodality therapy with neoadjuvant chemotherapy, total mastectomy with axillary lymph node dissection (ALND), and postmastectomy radiation therapy. This approach is associated with 5-year overall survival rates of 46% to 51%, with trimodality therapy being a statistically significant predictor of overall survival.^5,6,7^ ALND is recommended for all patients with IBC regardless of clinical nodal status or response to neoadjuvant chemotherapy.^8^ While sentinel lymph node biopsy (SLNB) is increasingly used in noninflammatory breast cancer, IBC has a unique clinicopathology characterized by invasion into the dermal lymphatics that limits the applicability and suitability of SLNB. Specifically, studies of the use of SLNB in IBC show high rates of failed SLN mapping and high false-negative rates compared with other forms of locally advanced breast cancer.^9,10,11^ However, whether and how SLNB is being used in clinical practice is not well described. In this study, we examine the frequency and temporal trend of SLNB in patients with IBC in the US.

## Methods

This cohort study followed Strengthening the Reporting of Observational Studies in Epidemiology (STROBE) reporting guideline. The institutional review board of MD Anderson Cancer Center deemed this study exempt from review and waived informed consent because patient data were deidentified and publicly available.

### Study Cohort

**Figure 1.**
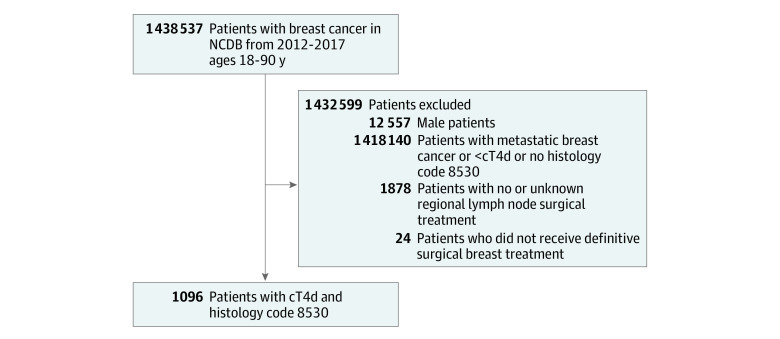
Cohort Selection Flowchart The histology code 8530 is from the *International Classification of Diseases for Oncology*. cT4d indicates clinical stage T4d; NCDB, National Cancer Database.

We used data from the National Cancer Database (NCDB), a nationwide hospital-based cancer registry representing approximately 70% of all new cancers diagnosed in the US. We identified female patients 18 years of age or older diagnosed with nonmetastatic IBC between 2012 and 2017. To examine patterns of care in IBC, we used a high-specificity definition of IBC, requiring both American Joint Committee on Cancer clinical stage T4d and *International Classification of Diseases for Oncology* (*ICD-O*) histology code 8530, to limit the inclusion of individuals misclassified as having IBC.^12^ We excluded patients with no (or unknown) regional lymph node surgery and those who did not receive definitive surgery (Figure 1).

### Study Variables

Demographic and clinical variables were examined as categorical and are defined in eTable 1 in the Supplement. Race and ethnicity were reported by participating hospitals and classified as Hispanic individuals, non-Hispanic Black individuals, non-Hispanic White individuals, and other, which included individuals of Asian, Native American, and unknown race or ethnicity. Race and ethnicity variables were collected and investigated as potential confounding factors. Regional lymph node surgery categories included SLNB only, SLNB followed by ALND, and ALND alone. As we sought to investigate any use of SLNB in IBC, we defined the variable any SLNB to include patients who underwent SLNB alone or SLNB followed by ALND. The variables and codes used to define each of these groups are summarized in eTable 2 in the Supplement.

### Statistical Analysis

We compared study variables between patients who underwent ALND alone and any SLNB using a χ^2^ test. We estimated the rate of any SLNB with 95% CIs overall and by year of diagnosis with the Cochran-Armitage trend test. To estimate the relationship between any SLNB rates and year of diagnosis, we constructed a scatterplot by fitting a linear regression model to evaluate the yearly increase of any SLNB use. We implemented univariable and multivariable logistic regression models to evaluate the association of study variables with the outcome of any SLNB. Multivariable model variables included those with a univariable association with any SLNB at *P* < .10 (ie, year of diagnosis, clinical nodal stage, chemotherapy, and the use of reconstructive surgery). Two-sided *P* values of <.05 were considered statistically significant. Statistical analyses were performed between March and June 2021 using SAS version 9.4 (SAS Institute).

## Results

**Figure 2.**
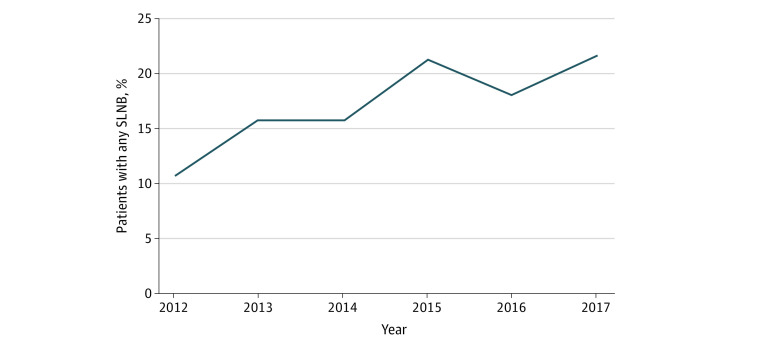
Rate of Any Sentinel Lymph Node Biopsy (SLNB) Use in Patients With Nonmetastatic Inflammatory Breast Cancer Over Time

Among 1 438 537 individuals in the NCDB with breast cancer from 2012 to 2017, 1096 women 18 years or older with nonmetastatic IBC were included in our analysis based on our prespecified criteria (mean [SD] age, 56.1 [12.9] years; 86 Hispanic women [7.8%]; 155 non-Hispanic Black women [14.1%]; 815 non-Hispanic White women [74.4%]) (Figure 1). Of the 1096 women included, 186 (17%) received any SLNB, and among individuals undergoing any SLNB, 119 of 186 (64%) did not undergo a completion ALND. Compared with those undergoing an ALND, individuals undergoing any SLNB had a later date of diagnosis, earlier clinical nodal stage (46 of 186 [24.7%] vs 124 of 910 [13.6%] at clinical node stage 0), and were more likely to undergo a partial mastectomy (10 of 186 [5.4%] vs 11 of 910 [1.2%]) (eTable 1 in the Supplement). We observed a statistically significant increasing trend in the use of SLNB from 2012 to 2017 with 22 of 205 patients with IBC (11%) undergoing an SLNB in 2012 and 32 of 148 (22%) in 2017 (*P* = .004) (Figure 2). The use of adjuvant radiation or trimodality therapy did not change during this time (eFigure 1 and eFigure 2 in the Supplement). Multivariable logistic regression analysis demonstrated that the use of SLNB was significantly associated with diagnosis year (2017 vs 2012; odds ratio [OR], 2.26; 95% CI, 1.26-4.20), clinical nodal status (cN3 vs 0; OR, 0.39; 95% CI, 0.22-0.67), and receipt of reconstructive surgery (OR, 1.80; 95% CI, 1.09-2.96) (Table).

**Table.** Multivariable Logistic Regression for Odds of Undergoing Any Sentinel Lymph Node Biopsy for All Patients (N = 1096)

Variable	OR (95% CI)	*P* value
Year of diagnosis		
2012	1 [Reference]	
2013	1.59 (0.87-2.89)	.13
2014	1.52 (0.79-2.75)	.22
2015	2.31 (1.22-3.88)	.009
2016	1.83 (1.00-3.12)	.05
2017	2.26 (1.26-4.20)	.006
Clinical nodal stage		
0	1 [Reference]	
1	0.54 (0.35-0.81)	.003
2	0.28 (0.16-0.52)	<.001
3	0.39 (0.22-0.67)	<.001
Unknown	0.25 (0.03-2.06)	.20
Chemotherapy		
Neoadjuvant	1 [Reference]	
Adjuvant	1.18 (0.41-3.36)	.76
None	0.40 (0.16-1.03)	.06
Reconstructive surgery		
No	1 [Reference]	
Yes	1.80 (1.09-2.96)	.02

Abbreviation: OR, odds ratio.

## Discussion

Our analysis suggests that SLNB is being frequently used in the management of IBC in the US despite current guidelines designating ALND as the only appropriate axillary surgical management option. Moreover, we found that the use of SLNB in patients with IBC was statistically and clinically significantly increasing over time. Our findings suggest a non–data-driven frequent and increasing use of SLNB in patients with IBC.

IBC is an aggressive form of invasive breast cancer with a high incidence of axillary node involvement and has double the mortality rate of noninflammatory locally advanced breast cancer.^4^ Randomized studies^13,14,15^ establishing the safety and use of SLNB in select patients with invasive breast cancer did not examine the use of SLNB in patients with IBC. This is highly relevant as the pathophysiology of IBC results in obstructed subdermal lymphatics that may impact normal nodal drainage and, therefore, result in false-negative rates with SLNB of up to 25%.^9,10^ Additionally, a prospective trial using dual tracer mapping found that SLN mapping was unsuccessful in 12 of 16 patients (75%) with IBC.^11^ While safe de-escalation of care in appropriately selected patients remains an important topic in breast cancer research, ALND has remained the standard of care in patients with IBC given the evidence that SLNB is not an effective technique in this patient population.

### Limitations

This study has limitations. First, our analysis was retrospective and susceptible to unaccounted bias and confounding sources. Second, we could not deduce the type of SLN mapping procedure (ie, dye, radiotracer, or both), whether the mapping was successful, or the reasons for completion ALND following SLNB. Third, given the limited clinical outcome data available, short-interval follow-up, and propensity for a selection bias related to the use of SLNB in more favorable individuals, we were unable to reliably explore clinical outcomes secondary to the use of SLNB. Finally, as our goal was to determine the use of SLNB in patients with IBC, we used a highly specific definition of IBC in our analysis: the presence of both American Joint Committee on Cancer clinical stage T4d and *ICD-O* pathologic code 8530.^16,17,18^ However, as pathologic evidence of IBC is not required for a diagnosis of IBC, this approach will exclude some individuals who have IBC from our analysis.

## Conclusions

In this study, we used hospital-based cancer registry data representing the majority of all new cancers diagnosed in the US to demonstrate a frequent and increasing use of SLNB in patients with IBC. The use of SLNB in patients with IBC is not evidence-based, not supported by current guidelines, and may represent an unsafe de-escalation of care in highly aggressive cancer. In the absence of high-quality randomized data, SLNB should not be used for patients with IBC.

## Notes

Supplement. **eTable 1.** Demographic and Clinical Variables of the Study Population **eTable 2. **Regional Lymph Node Surgery Codes **eFigure 1. **Rate of Any Adjuvant Radiation Use in Patients With Nonmetastatic Inflammatory Breast Cancer Over Time **eFigure 2. **Rate of Trimodality Therapy Use in Patients With Nonmetastatic Inflammatory Breast Cancer Over Time
