# Supplementary material for: Trends in Sentinel Lymph Node Biopsies in Patients With Inflammatory Breast Cancer in the US
Source: JAMA Netw Open. 2022 Feb 11;5(2):e2148021. doi: 10.1001/jamanetworkopen.2021.48021 (PMC8837909; doi:10.1001/jamanetworkopen.2021.48021)
Supplement: Supplement. — eTable 1. Demographic and Clinical Variables of the Study Population eTable 2. Regional Lymph Node Surgery Codes eFigure 1. Rate of Any Adjuvant Radiation Use in Patients With Nonmetastatic Inflammatory Breast Cancer Over Time eFigure 2. Rate of Trimodality Therapy Use in Patients With Nonmetastatic Inflammatory Breast Cancer Over Time [file jamanetwopen-e2148021-s001.pdf]

Supplemental Online Content

Sosa A, Lei X, Woodward WA, et al. Trends in sentinel lymph node biopsies in patients with inflammatory breast cancer in the US. *JAMA Netw Open*. 2022;5(2):e2148021. doi:10.1001/jamanetworkopen.2021.48021

eTable 1. Demographic and Clinical Variables of the Study Population
eTable 2. Regional Lymph Node Surgery Codes
eFigure 1. Rate of Any Adjuvant Radiation Use in Patients With Nonmetastatic Inflammatory Breast Cancer Over Time
eFigure 2. Rate of Trimodality Therapy Use in Patients With Nonmetastatic Inflammatory Breast Cancer Over Time

This supplemental material has been provided by the authors to give readers additional information about their work.

© 2022 Sosa A et al. *JAMA Network Open.*

eTable 1. Demographic and Clinical Variables of the Study Population

Variable	No. (%) ALND only N = 910	Any SLNB N = 186	*p* value
Age			
18-44	171 (18.8)	32 (17.2)	0.45
45-54	226 (24.8)	53 (28.5)	
55-64	270 (29.7)	55 (29.6)	
65-74	167 (18.4)	37 (19.9)	
75+	76 (8.4)	9 (4.8)	
Race			
Non-Hispanic Black	129 (14.2)	26 (14)	1
Hispanic	72 (7.9)	14 (7.5)	
Non-Hispanic White	676 (74.3)	137 (73.7)	
Other*	33 (3.6)	7 (3.8)	
Comorbidities (Charlson-Deyo Score)			
Score 0	743 (81.6)	157 (84.4)	0.65
Score 1	125 (13.7)	21 (11.3)	
Score 2+	42 (4.6)	8 (4.3)	
Insurance			
Private	478 (52.5)	106 (57)	0.44
Medicaid	137 (15.1)	26 (14)	
Medicare	233 (25.6)	41 (22)	
Other	18 (2)	1 (0.5)	
No insurance	32 (3.5)	10 (5.4)	
Unknown	12 (1.3)	2 (1.1)	
Education			
Level 1	156 (17.1)	27 (14.5)	0.33
Level 2	197 (21.6)	50 (26.9)	
Level 3	268 (29.5)	45 (24.2)	
Level 4	180 (19.8)	42 (22.6)	
Unknown	109 (12)	22 (11.8)	
Income			
Level 1	144 (15.8)	29 (15.6)	0.95
Level 2	194 (21.3)	36 (19.4)	
Level 3	203 (22.3)	46 (24.7)	
Level 4	260 (28.6)	53 (28.5)	
Unknown	109 (12)	22 (11.8)	
Area			
Metro	733 (80.5)	156 (83.9)	0.4
Non-metro	155 (17)	28 (15.1)	
Unknown	22 (2.4)	2 (1.1)	
**Variable**	**ALND only N = 910**	**Any SLNB N = 186**	***p* value**

© 2022 Sosa A et al. *JAMA Network Open.*

Year of Diagnosis			
2012	183 (20.1)	22 (11.8)	0.05
2013	160 (17.6)	30 (16.1)	
2014	133 (14.6)	25 (13.4)	
2015	140 (15.4)	38 (20.4)	
2016	178 (19.6)	39 (21)	
2017	116 (12.7)	32 (17.2)	
Histology Grade			
Grade 1	16 (1.8)	6 (3.2)	0.08
Grade 2	217 (23.8)	52 (28)	
Grade 3+	490 (53.8)	82 (44.1)	
Unknown	187 (20.5)	46 (24.7)	
Hormone Receptor Status			
HR+	305 (33.5)	58 (31.2)	0.48
HER2+	358 (39.3)	77 (41.4)	
TN	217 (23.8)	41 (22)	
Unknown	30 (3.3)	10 (5.4)	
Clinical Nodal Stage			
cN0	124 (13.6)	46 (24.7)	<0.001
cN1	448 (49.2)	95 (51.1)	
cN2	169 (18.6)	19 (10.2)	
cN3	158 (17.4)	25 (13.4)	
Unknown	11 (1.2)	1 (0.5)	
Chemo Type			
Neoadjuvant	831 (91.3)	176 (94.6)	0.19
Adjuvant	18 (2)	5 (2.7)	
Chemo - Timing unknown	1 (0.1)	0 (0)	
No chemo	60 (6.6)	5 (2.7)	
Surgery Type			
Total Mastectomy	899 (98.8)	176 (94.6)	<.001
Partial Mastectomy	11 (1.2)	10 (5.4)	
Radiation			
No	182 (20)	40 (21.5)	0.64
Yes	728 (80)	146 (78.5)	
Reconstructive Surgery			
No	833 (91.5)	161 (86.6)	0.033
Yes	77 (8.5)	25 (13.4)	
Lymphovascular Invasion			
No	239 (26.3)	51 (27.4)	0.86
Yes	451 (49.6)	88 (47.3)	
Unknown	220 (24.2)	47 (25.3)	
(continued)	**ALND only N = 910**	**Any SLNB N = 186**	***p* value**
Pathologic Nodal Stage			
pN0	339 (37.3)	71 (38.2)	0.006

© 2022 Sosa A et al. *JAMA Network Open.*

pN1	179 (19.7)	56 (30.1)	
pN2	212 (23.3)	30 (16.1)	
pN3	128 (14.1)	17 (9.1)	
Unknown	52 (5.7)	12 (6.5)	
Breast and Nodal pCR			
No	688 (75.6)	133 (71.5)	0.5
Yes	176 (19.3)	42 (22.6)	
Unknown	46 (5.1)	11 (5.9)	
Breast pCR			
n/a	88 (9.7)	19 (10.2)	0.37
No	599 (65.8)	116 (62.4)	
Yes	223 (24.5)	51 (27.4)	
Nodal pCR			
n/a	52 (5.7)	12 (6.5)	0.75
No	519 (57)	103 (55.4)	
Yes	339 (37.3)	71 (38.2)	
Miles to Hospital			
0-10 miles	419 (46)	72 (38.7)	0.19
11-30 miles	338 (37.1)	79 (42.5)	
31+ miles	153 (16.8)	35 (18.8)	
Facility Type			
Community Cancer Program	117 (12.9)	23 (12.4)	0.33
Comprehensive Cancer Center	367 (40.3)	67 (36)	
Academic Center	234 (25.7)	53 (28.5)	
Integrated Center	93 (10.2)	27 (14.5)	
Unknown	99 (10.9)	16 (8.6)	

*Asian, Native American, and Unknown

© 2022 Sosa A et al. *JAMA Network Open.*

eTable 2. Regional Lymph Node Surgery Codes

	SLNB only (N = 119)	SLNB + ALND (N = 67)	ALND only (N = 910)
**RX_SUMM_SCOPE_REG_LN_2012**	02 – Sentinel Lymph Node Biopsy	06 – Sentinel Lymph Node Biopsy → completion dissection (same day)<br>07 – Sentinel Lymph Node Biopsy → completion dissection (different days)	03 – Regional Lymph Node Dissection (# of nodes unknown)<br>04 – Regional Lymph Node Dissection (1-3 nodes removed)<br>05 – Regional Lymph Node Dissection (4+ nodes removed)

© 2022 Sosa A et al. *JAMA Network Open.*

eFigure 1. Rate of Any Adjuvant Radiation Use in Patients With Nonmetastatic Inflammatory Breast Cancer Over Time

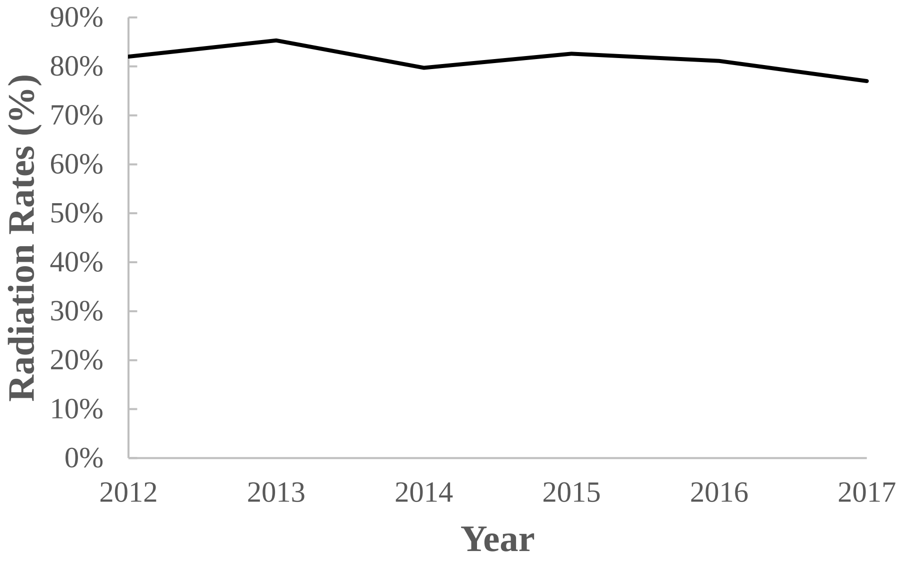

© 2022 Sosa A et al. *JAMA Network Open.*

eFigure 2. Rate of Trimodality Therapy Use in Patients With Nonmetastatic Inflammatory Breast Cancer Over Time[a]

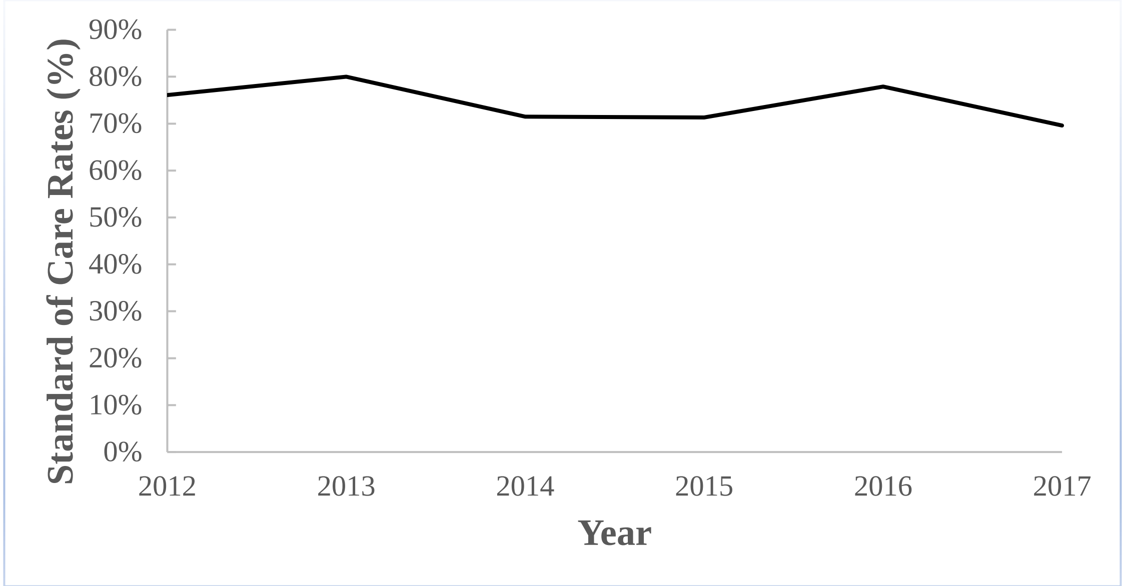

[a]Trimodal therapy includes chemotherapy, surgery, and radiation.

© 2022 Sosa A et al. *JAMA Network Open.*
